# Taxonomic, structural diversity and carbon stocks in a gradient of island forests

**DOI:** 10.1038/s41598-022-05045-w

**Published:** 2022-01-20

**Authors:** Lurdes C. Borges Silva, Diogo C. Pavão, Rui B. Elias, Mónica Moura, Maria A. Ventura, Luís Silva

**Affiliations:** 1 CIBIO, Centro de Investigação em Biodiversidade e Recursos Genéticos, InBIO Laboratório Associado, Pólo dos Açores, Universidade dos Açores, Campus de Ponta Delgada, Rua da Mãe de Deus, 9500-321 Ponta Delgada, Açores, Portugal; 2 Faculdade de Ciências E Tecnologia, Universidade Dos Açores, Campus de Ponta Delgada, Rua da Mãe de Deus, 9500-321 Ponta Delgada, Açores, Portugal; 3 CE3C/ABG – Centre for Ecology, Evolution and Environmental Changes/Azorean Biodiversity Group, Universidade dos Açores, Campus de Angra do Heroísmo, Rua Capitão João d’Ávila – Pico da Urze, 9700-042 Angra do Heroísmo, Portugal; 4 Faculdade de Ciências Agrárias e do Ambiente, Universidade dos Açores, Campus de Angra do Heroísmo, Rua Capitão João d’Ávila – Pico da Urze, 9700-042 Angra do Heroísmo, Portugal

**Keywords:** Biodiversity, Ecosystem ecology, Ecosystem services, Forest ecology, Forestry, Ecosystem ecology, Ecosystem services, Forest ecology, Forestry

## Abstract

Assessment of forest ecosystems and their services is seen as a key action for the advancement of biodiversity objectives, and to inform the development and implementation of related policies and planning. The Azorean forest is important for timber production, the protection of soil and water resources, and for its recreational and aesthetic value. However, its role in carbon accumulation has not been fully addressed. We assessed plant diversity, forest structure and carbon stocks in a gradient of three forest types (Natural Forest-NF; Exotic Woodland-EW and Production Forest-PF) in three of the Azores islands. We used biodiversity indices and found that NF harbored the highest plant diversity levels and PF the lowest. Diversity levels were lower for structural than for taxonomic data, particularly for PF. The highest tree carbon stock was found at EW in one of the islands, while PF consistently exhibited relatively high tree carbon stocks in the three islands. The largest soil carbon stocks were found at EW, while leaf litter carbon stocks were higher at PF. We concluded that NF play a fundamental role as plant diversity hotspots but have lower relevance as carbon stocks what might be associated with montane environmental conditions. PFs provide economic assets and act as carbon sinks, while EWs play a major role as carbon sinks in soil, but also at tree level in the oldest forests.

## Introduction

Forests are one of the richest ecosystems^1^ providing a wide range of products and ecosystem services with vital importance to the functioning of the biosphere^2–4^. Thus, forests have become increasingly prominent on the international policy and scientific agenda, receiving attention from scientists in various fields of research and from policymakers^4–6^.

Forests support biodiversity maintenance and conservation^1,7,8^, and given the multitude of ecosystem services provided, it is difficult to generalize the overarching role of forests on biodiversity^1,9,10^. Nevertheless, there has been much progress on the understanding of the effects of forest biodiversity on single and multiple ecosystem services, with the number of published reviews signaling that this is a very active field of research^1,9,10^.

The relationships between forest type, biodiversity conservation and ecosystem services are highly relevant for informing forest policy and management^1,2^. Meanwhile, there is considerable evidence that natural forests may harbor higher plant diversity levels (e.g., natural tropical forests could have between 70 and 300 or more tree species per hectare) than forest plantations (one or two tree species at planting)^11^. Hence, the most important characteristics of tropical and subtropical humid forests are their species richness, heterogeneity, and complex community organization^12,13^. Other research has shown that temperate forests are also plant diversity hotspots with high levels of endemism^14^, being highly diverse in species, including soil organisms, playing a relevant role as carbon sinks^6,15,16^, and providing important ecosystem services globally, regionally, and locally^14,17,18^.

The carbon pools and fluxes in forest ecosystems are affected by many different factors, such as species diversity and identity, tree growth, understory vegetation, leaching of dissolved organic matter, and organic matter decomposition rate^19–21^. According to several studies^22–24^, not all forests have the same capacity to capture and store carbon. Madrigal-González et al.^25^, sampling natural forests on five continents, showed that forests located in cold or dry regions, and the abundance of trees, favour the recapture of CO_2_. Kendie et al.^26^, after comparing biomass and soil carbon stock potential between natural forests, *Eucalyptus* plantations and regenerated secondary forests, concluded that the carbon pool variation was significant, and that natural forests stored a higher amount of carbon, thus playing an important role in climate change mitigation. Others have shown that indigenous forests sequester more carbon in biomass and soil than did 30 to 50-year-old plantations of exotics, but it remains unclear if this was an intrinsic difference between both types or a difference resulting because of insufficient time for soil organic carbon levels in plantations to recover after the clearance of the original indigenous forest^27^.

Productivity in terrestrial ecosystems is directly linked to nutrient cycling among the various components of the plant-soil system^28,29^. In forest ecosystems, primary production is usually evaluated through litter production because litter is the main source of soil organic carbon and plant nutrient cycling^30,31^, although some soils nutrients reflect their concentration in plants rather than in litter^32,33^. However, litter production and decomposition in forests can vary with forest type, climate, and ecosystem disturbance (e.g., planted species showed a stronger influence on rates of decomposition and soil respiration than indigenous species)^29,31^.

To date, the ecosystem services concept has so far received little attention in islands around the globe^34^. These areas, including temperate, semi-tropical or tropical forests, are rich in biodiversity and natural resources, and provide a variety of ecosystem services of global and regional importance (e.g., water regulation, erosion control, pollination, pest-control, food supply and recreation)^34^. This translates to a substantial but often unrecognized contribution to local island economies, crucial contributions to the tourism sector, many cultural ecosystem services depending on indigenous diversity and healthy ecosystems^34^.

To improve the understanding of ecosystem processes and investigate relationships between biodiversity and ecosystem function, islands have been treated as ‘model systems’^35,36^. A broad range of ecological studies have used island as models, (e.g., Mauritius, Krakatau, Hawaii, Galapagos, Madagascar, New Zealand, and Australia), in order to better understand ecological and evolutionary processes^35,36^. Some results indicate that there is a significant relationship between island area and plant species composition^37^. This relationship was found to be a major factor in determining several ecosystem-level properties of these islands, including standing biomass, plant litter decomposition, nitrogen mineralization, terrestrial carbon partitioning, humus accumulation, and plant nitrogen acquisition^35,38^.

The Azores archipelago is an interesting region to be used as a model in studies devoted to plant diversity pattern changes, associated with anthropogenic activity, and to the potential ecosystem services originated by different forest types. In fact, Azorean forests have an important role in the conservation of water resources and in the refilling of aquifers^39^, since the large areas of pastureland tend to have an impermeable layer relatively close to the soil surface, leading to increased water runoff and to decreased infiltration^40^, while forests contribute to precipitation and occult precipitation interception, which is very common in the Azores^41,42^. Moreover, this also contributes to avoid soil erosion^43,44^ due to torrential discharges and earthquakes, which are common in the Archipelago throughout the year^45^. Forests also play an important role in recreation areas for the local population through the network of recreational forest areas^46^, but also through the extensive network of hiking trails which crosses different forest types being an important tourism resource^47^. Moreover, the production forest dedicated to *C. japonica* is presently responsible for 1400 jobs, and for a revenue of 12 million euros annually^48^.

However, the knowledge about ecosystem services in the Azores is still quite limited and only a few studies have been published. Those studies showed that there is a considerable loss of plant diversity associated with the impact of anthropogenic disturbance across a landcover gradient of community types^49,50^. To quantify forest carbon sequestration in the Azores Islands, some studies have estimated the total carbon stocks for exotic species (e.g., *Cryptomeria japonica, Pittosporum undulatum*)^51,52^. A remote assessment of changes in carbon storage on Pico Island (Azores, Portugal), indicated that an increase in carbon stocks (economical value) while protecting biodiversity (environmental value), would be possible through adapted and synergic management actions^52^. Other research showed that a considerable amount of woody biomass is available in the Azores islands with private companies interested in the use of forest residues^53–55^. For native forests, studies showed that carbon sequestration was related to productivity and vascular plant diversity^56^. Other studies have quantified socioeconomic benefits derived from natural forests and demonstrated that nature conservation and biodiversity areas can drastically improve quality of life and economic self-sufficiency of local populations by the diversification and creation of new skills, products and business opportunities^57,58^.

Nevertheless, a comparison of taxonomic diversity values, structural diversity, and carbon accumulation, based on a detailed collection of field data, and addressing the three main forest types in the Azores—production forest (PF), exotic woodland (EW), and natural forest (NF)—has not been attempted. Therefore, the main goal of this research was to determine the ecosystem services presently associated with the forest areas in the Azores, with particular attention to plant diversity (all vascular plants, including trees, shrubs, herbaceous plants, and ferns) and carbon stocks. Based on previous research dedicated to each forest type, we hypothesized that: (i) the highest levels of taxonomic diversity would be found in natural forest stands; (ii) the diversity levels would be lower for structural than for taxonomic data; (iii) tree carbon stocks would be larger in production forests; (iv) leaf litter and soil carbon stocks would be larger in natural forests which are less disturbed; and (v) larger differences would be found between forest types than between islands. Thus, our specific objectives for the three main forest types were: (i) to evaluate the taxonomic diversity; (ii) to evaluate the structural diversity using a dendrometric approach; (iii) to evaluate the carbon stocks in standing biomass using allometric equations; and (iv) to evaluate the carbon stocks in leaf litter and soil using chemical analyses.

Although this study focused on a single region, findings can be used as a model for other forests, regions and small islands given commonalities in size, natural resources, and ecosystems^35,36^.

## Results

### Taxonomic diversity

Globally, the three forest types in São Miguel showed the largest number of vascular taxa, although with the largest proportion of exotic elements, while the forest in Pico showed the highest contribution of endemic and native taxa (Table 1).

**Table 1:** Plant species richness at the three types of forests sampled on three islands in the Azores archipelago.

Island	Taxa						
	Endemic		Native		Exotic		Total
	N	%	N	%	N	%	
São Miguel	24	30	24	30	32	40	74
Terceira	20	41	17	35	12	24	45
Pico	30	44	26	38	12	18	62

Endemic taxa only occurring in the Azores; Native taxa that colonized the Azores without human intervention, also occurring in other regions; and Exotic taxa that were intentionally or accidentally introduced by human activities^59^.

For *α* diversity there was only a significant effect of forest type (F = 152.09; p < 0.01), NF differing from EW and PF (Table 2). Regarding *β* diversity, there was a significant effect of island (F = 8.48; p < 0.01) and forest type (F = 51.90; p < 0.01), with the largest values at NF, particularly in Pico and São Miguel, and with a relatively high value at the EW in São Miguel (Table 2). There was a significant effect of island (F = 5.88; p < 0.01) and forest type (F = 151.55; p < 0.01) on γ diversity, with the highest values at NF (Table 2). Again, there was a significant effect of island (F = 7.71; p < 0.01) and forest type (F = 146.50; p < 0.01) on Shannon diversity, with the highest values in NF (Table 2). In terms of evenness, there was a significant effect of forest type (F = 42.30; p < 0.01), but not of island (F = 1.57; p = 0.21), NF showing the highest levels, followed by EW (Table 2).

**Table 2:** Plant taxonomic diversity found at 90 forests in the Azores, from three islands (Pico, São Miguel, and Terceira) and three forest types (Exotic Woodland, Natural Forest, and Production Forest).

Diversity		Exotic Woodland						Natural Forest						Production Forest					
		Pico		São Miguel		Terceira		Pico		São Miguel		Terceira		Pico		São Miguel		Terceira	
α	m	4.8	a	7.1	a	3.8	a	20.6	b	16.8	b	16.3	b	4.2	a	5.7	a	3.4	a
	se	0.5		0.8		0.3		1.2		2.6		0.6		0.5		0.7		0.4	
β	m	1.8	a	4.2	bcd	1.6	a	6.9	e	6.4	de	4.9	cde	2.2	ab	3.1	abc	2.4	ab
	se	0.3		0.6		0.3		0.3		0.9		0.2		0.3		0.6		0.5	
γ	m	6.6	a	11.3	a	5.4	a	27.5	c	23.2	bc	21.2	b	6.4	a	8.8	a	5.8	a
	se	0.8		1.6		0.4		1.4		3.1		0.5		0.7		1.2		0.9	
H	m	1.5	abc	1.9	c	1.3	ab	3.0	d	2.8	d	2.8	d	1.3	ab	1.6	bc	1.1	a
	se	0.1		0.1		0.1		0.1		0.2		0.0		0.1		0.2		0.1	
E	m	0.8	cd	0.8	bcd	0.8	abc	0.9	d	0.9	d	0.9	d	0.7	ab	0.8	abc	0.7	a
	se	0.0		0.0		0.0		0.0		0.0		0.0		0.0		0.0		0.0	

Alpha, beta and gamma diversities, Shannon diversity (H) and Evenness (E). Mean and standard error for each diversity parameter. For each row, different letters indicate significant differences (p < 0.05) according to the results of a Tukey test applied after ANOVA.

Concerning hierarchical diversity partitioning, *α*_1_ diversity represented about 9% of the total species diversity, *α*_2_ 13%, and *α*_3_ 55% (Table 3). According to *β* diversity results, differentiation among plots within the same forest was relatively low (*β*_*1*_), while there was an important component of differentiation among forests within the same type (*β*_*2*_) and among forest types (*β*_*3*_) (Table 3).

**Table 3:** Partitioning of plant diversity, according to the hierarchical model of additive partitioning.

Diversity	α_1_	α_2_	α_3_	β_1_	β_2_	β_3_	γ
Value	10.2	13.9	59.0	3.7	45.1	49.0	108.0
%	9.4			3.5	41.7	45.4	100.0
%		12.9			41.7	45.4	100.0
%			54.6			45.4	100.0

Partition of γ diversity into its α and β components at three spatial scales (i) among plots within the same forest; (ii) among forests within the same type; and (iii) among forest types.

### Forest clusters

**Figure 1:**
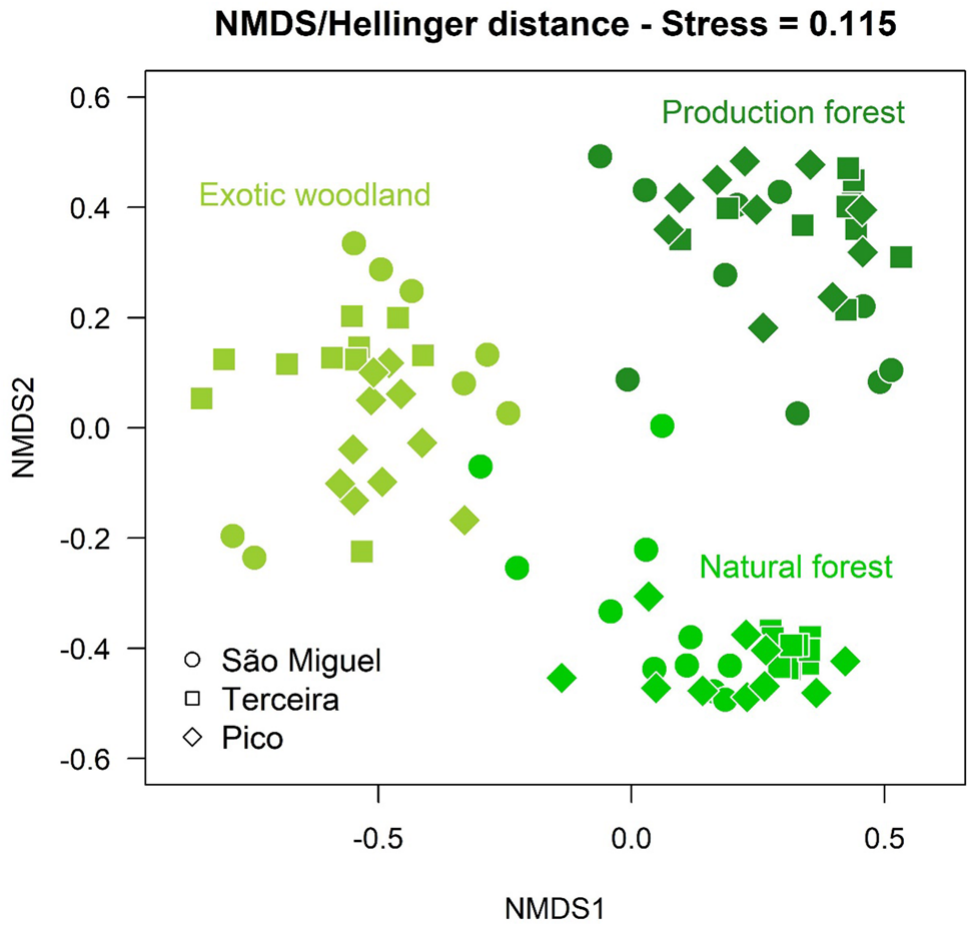
Non-Metric Multidimensional Scaling applied to the Hellinger distance matrix, based on plant species abundances, and applied to all forest types. The three colors represent the three community types obtained by using numerical ecology methods: Natural Forest, Exotic Woodland and Production Forest.

Based on the value of cophenetic correlation, the best combination of distance metric and agglomeration algorithm corresponded to Hellinger Distance and UPGMA. The NMDS resulting from the projection of Hellinger distances showed the 90 forests assembled into three groups (Fig. 1), each associated with different dominant species: EW—*P. undulatum* and *A. melanoxylon*; PF—*C. japonica* and *H. macrophylla*; NF—*L. azorica*, *I. azorica* and *J. brevifolia*.

The PERMANOVA confirmed that forest type explained the largest proportion (64%) of the variation in the vascular plant cover dataset, while island explained only 2%. The indicator species analysis also confirmed the expected differences between forests (see Supplementary Table S1 online): NF—17 species with significant indicator value; EW and PF only 2 and 1 indicator species, respectively. *Vaccinium cylindraceum* (95%), *M. africana* (95%) and *I. azorica* (93%) showed the highest indVals for NF. In addition, several indigenous species were present in NF, including trees/shrubs and ferns. The IndVals for *Pittosporum undulatum* (92%) and *Cryptomeria japonica* (99%) were the highest, respectively for EW and PF.

### Environment and soil parameters

There was a significant difference between forest types regarding altitude (F = 68.15; p < 0.01), with a tendency for EW being located at lower elevations and NF at the highest (Table 4).

**Table 4:** Environmental variables found at 90 forests in the Azores, from three islands (Pico, São Miguel, and Terceira) and three forest types (Exotic Woodland, Natural Forest, and Production Forest).

		Exotic Woodland						Natural Forest						Production Forest					
		Pico		São Miguel		Terceira		Pico		São Miguel		Terceira		Pico		São Miguel		Terceira	
ALT (m)	m	272.4	a	349.8	ab	202.2	a	683.8	de	616.2	cde	711.1	e	491.7	bc	538.3	cde	507.9	bcd
	se	13.7		44.6		37.6		57.7		33.2		33.5		47.9		55.0		35.4	
TMEA (ºC)	m	16.7	d	15.2	bc	16.5	cd	13.8	ab	13.1	a	13.0	a	15.1	bc	13.6	a	14.3	ab
	se	0.3		0.3		0.4		0.4		0.3		0.2		0.4		0.4		0.2	
PMEA (mm)	m	2059.1	abc	1644.8	ab	1254.5	a	3117.1	d	2616.5	cd	2695.4	cd	2732.7	cd	2349.5	bcd	2208.6	bc
	se	214.1		159.7		82.7		267.5		158.9		135.3		249.1		179.8		116.3	
RHMEA (%)	m	87.7	a	91.8	b	90.0	ab	95.4	cd	95.8	cd	97.9	d	92.4	bc	95.5	cd	95.8	cd
	se	1.1		0.7		0.9		0.8		0.6		0.3		1.1		1.0		0.3	

Mean and standard error for each environmental parameter. For each row, different letters indicate significant differences (p < 0.05) according to the results of a Tukey test applied after ANOVA.

There was a significant difference between islands and forest types for mean annual temperature (TMEA; F = 11.00, p < 0.01; F = 59.40, p < 0.01), mean annual relative humidity (RHMEA; F = 10.95, p < 0.01; F = 53.72, p < 0.01) and mean annual precipitation (PMEA; F = 8.22, p < 0.01; F = 31.18, p < 0.01). Lowest temperatures were found at NF, followed by PF (Table 4). Regarding relative humidity and precipitation, this pattern was reversed (Table 4).

Concerning soil parameters, significant differences between islands and forest types are reported (see Supplementary Table S2 online). Bulk density was lower in Pico while OM and total N tended to be high in that island (see Supplementary Table S3 online). The values of pH tended to be the lowest at NF, while Ca, Mg and Na values appeared to be larger in Pico and at EW; the values of P, K and Al appeared to be somewhat irregular (see Supplementary Table S3 online). Soil texture was mostly dominated by loam or sandy soil, with no differences between forest types (see Supplementary Fig. S1 online). However, differences were obtained between Pico, where forest soils were mostly composed of basaltic rock and sandy loam, and the other two islands.

### Structural diversity

Regarding structural diversity, significant differences between islands and forest types are reported on Table 5. EW tended to show the highest tree density, while PF showed the lowest (Table 6). In general, PF showed the highest basal areas per hectare (Table 6), except for some stands of EW in São Miguel Island. The number of taxa contributing to *BA* (*BA* γ) was much larger at NF, followed by EW (Table 6). Likewise, structural diversity indices for *BA* (*BA H*, *BA E*) were significantly higher in NF, and lowest at PF (Table 6). PF showed the highest average tree heights (Table 6). Mean *AGB* was highest for EW stands in São Miguel Island and lowest for NF. However, PF showed, consistently and for all islands, high values of *AGB* (Table 6).

**Table 5:** Comparison of structural diversity at 90 forests in the Azores, from three islands (Pico, São Miguel, and Terceira) and three forest types (Exotic Woodland, Natural Forest, and Production Forest).

Parameter	Type		Island		Interaction	
	F	p	F	p	F	p
D	18.8	**0.000**	2.7	0.072	0.5	0.762
BA	79.5	**0.000**	1.2	0.304	3.6	**0.009**
BA γ	99.9	**0.000**	2.0	0.144	0.9	0.495
BA H	61.3	**0.000**	4.5	**0.014**	1.3	0.277
BA E	49.2	**0.000**	3.1	0.052	0.6	0.684
H	254.2	**0.000**	3.2	**0.046**	5.1	**0.001**
AGB	6.1	**0.003**	7.2	**0.001**	7.8	**0.000**

Tree density (D, trees ha^−1^), Basal area (BA, m^2^), BA γ (number of taxa contributing to the BA, i.e., those with a diameter at breast height above 2.5 cm), Shannon diversity based on BA (BA H), Evenness based on BA (BA E), aboveground biomass (AGB Mg ha^−1^). Results of a two-way ANOVA. Bold indicates a significant effect (p < 0.05).

**Table 6:** Structural diversity found at 90 forests in the Azores, from three islands (Pico, São Miguel, and Terceira) and three forest types (Exotic Woodland, Natural Forest, and Production Forest).

Parameters		Exotic Woodland						Natural Forest						Production Forest					
		Pico		São Miguel		Terceira		Pico		São Miguel		Terceira		Pico		São Miguel		Terceira	
D (trees ha^−1^)	m	5310.0	c	4040.0	bc	3940.0	abc	3770.0	abc	3110.0	ab	3110.0	ab	2330.0	ab	1970.0	a	2130.0	ab
	se	734.9		836.6		454.2		367.0		331.1		165.0		202.2		214.5		265.9	
BA (m^2^)	m	0.6	a	0.9	a	0.5	a	0.5	a	0.5	a	0.5	a	1.9	bc	1.8	b	2.6	c
	se	0.1		0.2		0.1		0.1		0.1		0.1		0.3		0.3		0.3	
BA γ (taxa/plot)	m	3.1	b	2.4	ab	2.6	ab	6.5	c	5.3	c	5.4	c	1.2	a	1.3	a	1.3	a
	se	0.5		0.4		0.2		0.6		0.6		0.5		0.1		0.2		0.2	
BA H	m	0.6	bc	0.4	ab	0.5	bc	1.1	d	0.7	bc	0.9	cd	0.0	a	0.0	a	0.0	a
	se	0.1		0.1		0.1		0.1		0.1		0.1		0.0		0.0		0.0	
BA E	m	0.5	cd	0.3	bc	0.5	cd	0.6	d	0.4	cd	0.6	cd	0.0	ab	0.0	a	0.0	ab
	se	0.1		0.1		0.1		0.1		0.1		0.1		0.0		0.0		0.0	
H	m	8.8	ab	9.4	b	8.2	ab	5.1	ab	4.6	a	4.7	a	22.8	cd	18.9	c	26.3	d
	se	0.6		1.0		0.7		0.5		0.5		0.3		1.5		1.3		1.7	
AGB (Mg ha^−1^)	m	7.8	a	266.2	b	9.6	a	6.1	a	7.0	a	6.5	a	47.2	a	59.0	a	86.8	a
	se	1.5		91.2		3.3		1.5		1.6		0.7		5.9		16.6		14.6	

Mean and standard error for each structural parameter. Tree density (D, trees ha^−1^), Basal area (BA, m^2^), BA γ (number of taxa contributing to the BA, i.e., those with a diameter at breast height above 2.5 cm), Shannon diversity based on BA (BA H), Evenness based on BA (BA E), aboveground biomass (AGB Mg ha^−1^). For each row, different letters indicate significant differences (p < 0.05) according to the results of a Tukey test applied after ANOVA.

### Carbon stock in the trees

Total carbon storage in the trees (*AGB* and *BGB*) was significantly different between forest types (F = 7.559, p < 0.01) and between islands (F = 7.893, p < 0.01). The highest value was found at EW in São Miguel while the lowest was found at NF in the three islands (Table 7). For all islands, PF exhibited relatively high values of carbon stocks (Table 7).

**Table 7:** Carbon stock found at 90 forests in the Azores, from three islands (Pico, São Miguel, and Terceira) and three forest types (Exotic Woodland, Natural Forest, and Production Forest).

		Exotic Woodland						Natural Forest						Production Forest					
		Pico		São Miguel		Terceira		Pico		São Miguel		Terceira		Pico		São Miguel		Terceira	
C|AB	m	247.7	a	4569.4	b	185.5	a	147.4	a	133.2	a	145.6	a	526.8	a	475.0	a	797.1	a
	se	34.4		1472.3		23.5		34.9		22.9		14.1		56.9		82.4		87.2	
C|L	m	2.4	bc	1.3	ab	1.9	ab	1.6	ab	1.6	ab	0.9	a	3.6	c	2.1	ab	3.5	c
	se	0.2		0.1		0.3		0.3		0.2		0.4		0.5		0.2		0.4	
C|S	m	26.4	a	89.5	c	100.0	c	25.8	a	65.3	ab	76.1	c	30.4	a	65.1	ab	78.0	c
	se	2.8		10.1		15.1		5.7		113.0		15.3		3.9		5.0		7.5	

Mean and standard error for each structural parameter. Carbon stock in the trees, both above and below ground (C|AB, Mg ha^−1^), in leaf litter (C|L, Mg ha^−1^), and in soil (C|S, Mg ha^−1^). For each row, different letters indicate significant differences (p < 0.05) according to the results of a Tukey test applied after ANOVA.

### Carbon stock in the leaf litter and in soil

Regarding leaf litter, carbon stock was significantly different between forest types (F = 24.83, p < 0.01) and between islands (F = 5.98, p < 0.01); Pico Island showing higher stocks as well as PF (Table 7). Carbon stocks in soils were significantly different between islands (F = 29.92, p < 0.01) but not between forest types (F = 2.55, p = 0.08). In general, Pico Island soils showed the lowest values of carbon accumulation (Table 7).

## Discussion

Here we discuss taxonomic and structural diversity, and the carbon stock estimates obtained for the different compartments, for each forest type and island.

### Taxonomic diversity

NF plots showed higher species diversity than PF and EW plots, which agrees with previous studies for the Azores^49,50,56,60–62^, and other regions^63–65^. The low plant diversity noted in PF could be explained by the dominance of a single species (*Cryptomeria japonica*), contributing with nearly 90% of the total number of trees per plot, and dominating the canopy where only ferns and a few invasive species that tolerate low levels of light (e.g., *Hedychium gardnerianum*) are found^66,67^.

Moreover, these differences in plant diversity could also be explained by climatic conditions, soil type and anthropogenic action^61,68–73^. Concerning NF, our plots mainly corresponded to montane forests^61^ which occur in the thermotemperate-hyperhumid and ultrahyperhumid belts, from 600 to 1000 m a.s.l., in areas with high rainfall (3000 to 5000 mm year^−1^) and occult precipitation (cloud forests)^74,75^. These forests have small stature and are frequently subjected to natural disturbances^75^. This favors plant diversity by allowing the existence of both light-demanding and shade tolerant species^76^. They are characterized by a high percentage of endemic species, trees covered by epiphytes and a complex vertical structure with several layers^61^.

Regarding EW, the dominant tree *Pittospotum undulatum* grows in mild, humid and sub-humid climatic zones, with some growth limitation at higher elevations. Its dense canopy, tends to form a continuous layer with light intensities below 1% full sunlight at ground level^77^, leading to a reduction in plant diversity^78,79^.

Regarding anthropogenic action, NF have less human influence and are hard to access.^80^. Disturbances are limited to minor harvesting of non-timber forest products^65^. However, an intensive management regime in the case of PF and the spread of invasive species in EW, originated a decrease in plant diversity levels^80–84^. While plantations are known for high timber productivity, their potential to harbor plant diversity is low^72^. In the Azores, the new production forests already include a buffer zone with native elements^85^.

### Structural diversity

Our results showed differences among forest types in tree diameter, height, basal area, and forest density. The structure of PF and EW was mainly influenced by the respective dominant species. The structural dominance of *Pittosporum undulatum* in EW and of *Cryptomeria japonica* in PF confirm their potential to originate pure stands^53–55,73,85–89^.

NF showed the highest values for structural diversity, with larger number of woody taxa. Natural forests are often characterized by greater structural diversity^49,50,61,62,90–92^ than managed forests, where silvicultural practices often uniformize species composition and structure^93–97^. Species diversity increases structural diversity when different life strategies coexist and can also promote tree size and canopy height heterogeneity^98^, while structural diversity can be a proxy for species diversity^99^.

### Carbon stock in trees

The primary Azorean forest, which existed before the arrival of human settlers, was largely cut, having been replaced by secondary forest with the potential to sequester a large amount of carbon because of their rapid regrowth following disturbance^100,101^.

Our study revealed that, in São Miguel, *AGB* was highest in some of the EW stands, indicating the presence of old forests^54^, making *Pittosporum undulatum* an important carbon sink, as shown in previous studies^55^. As expected, PF showed high values^51^, and NF the lowest values of total carbon storage. The latter could be due to climatic and soil limitations, since large stature forests, although rare, are still present at sheltered low altitude locations in the Azores, but also to differences in tree age and growth rate, estimates of which are still lacking for most Azorean trees^102^.

Although previous studies in other regions suggest a larger accumulation of carbon in natural versus managed forest^103,104^ we have not confirmed this trend. Our data and previous analyses suggest that extant NF show relatively high tree densities, but smaller tree heights than PF, larger trees being restricted to the older forests. The small height in NF could be associated with environmental conditions in montane belts where trees with contorted trunks and branches, dense compact crowns, small and hard leaves are found^61^. Moreover, the submontane forest dominated by *Laurus azorica* has been mostly replaced by pastureland or exotic woodland^61,72,94^, the existing stands being limited in distribution range and in age, with many relatively young trees^102^, often being invaded by *P. undulatum* or *A. melanoxylon*^81,88^. One such examples is the *Laurus* dominated forest that we sampled at Povoação (São Miguel Island). Thus, presently, *Laurus* dominated forest has a reduced contribution to carbon accumulation in the Azores, at least in comparison with the theoretical potential, based on its climatically suitable distribution^61^. Other more thermophilic forests, mostly found at low elevations, such as *Morella* and *Picconia* woodland^61^, are only preserved in some of the islands, being represented by relatively small stands, frequently invaded by *P. undulatum*. Although these stands can attain a high stature (e.g., 15 m), their limited distribution makes their present contribution to carbon accumulation relatively low. It is clear, nonetheless, that if a carbon market is to be established in the Azores, natural forests could regain importance, since, as a surplus, they harbor huge amounts of native and endemic species of plants and animals^72^. Notwithstanding, for more precise estimates of carbon sequestration, their growth rate should be considered, making dendrochronological studies more relevant^102,105^.

A total 239.17 Mg ha^−1^ has been estimated for Terceira Island, approximately 75%, sequestered by *Juniperus brevifolia*, a value somewhat larger than our average results, but within the same order^56^. Based on *AGB* values for the Laurisilva in the Canaries islands, and assuming that sequestered carbon corresponds to 50% of the biomass^106,107^, a value of 127.55 Mg ha^−1^ has been estimated, which is very close to our estimates.

*Cryptomeria japonica* stands in the Azores are exploited under high shaft, with minimum revolutions of 30 years, and with an annual productivity that can exceed 20 m^3^ ha^−1^ year^−1^. The oldest and largest *Cryptomeria* stands are found in São Miguel Island with an average of 32 years^51^ and encompassing 70% of the archipelago's populations^108^. The total carbon stock present in the Azorean *Cryptomeria japonica* forest was estimated at 2816 ± 1594 Gg (in a total of 12,968 ha), representing about 217 Mg ha^−1^^51^, a value close to that found in our study. Fukuda et al.^109^, for an area of 4.51 × 10^6^ ha obtained values between 80.79 and 90.11 Mg ha^−1^. Similarly, Sasaki and Kim^110^ obtained values of 24.3 to 48.7 Mg ha^−1^, and of 76 to 101.6 Mg ha^−1^, for natural and planted forest, respectively.

### Carbon stock in leaf litter

Differences between forest types in leaf litter carbon stock were found in our study as seen in other studies^111,112^. The PF contained a high carbon concentration in leaf litter and a high accumulation of litter. Other forest types capable of significantly altering soil organic carbon stock in temperate forests have been reported^113,114^. Coniferous litter contains more lignin, which slows down the rate of decomposition, leading to more litter accumulation in the forest floor and the formation of acidic compounds^115,116^. In these acidic soils, soil fauna is less active, decreasing the amount of humus mixing through mineral soil and leaving more materials in the forest floor^117^. In addition, conifers have shallower rooting systems and tend to accumulate more organic carbon in the forest floor^118^. The humus profile is usually thinner in deciduous and sclerophyllous forests than in coniferous forests. The lower rate of decomposition of the leaf litter in *Cryptomeria japonica* plantations and *Pinus resinosa* forests, compared to those of oak forests, may be due to their chemical properties^119–121^. Although large amounts of litter may not increase carbon in soil, low litter inputs usually result in a rapid carbon soil decline^122^.

### Carbon stock in the soil

Our study showed that Pico Island soils are relatively young, mostly composed by basaltic rock debris (i.e., leptosols). The Azorean soils are mainly andosols (i.e., soils that have formed from volcanic ash or other volcanic ejecta)^123^. Soils in Pico Island are mostly formed by basaltic rocks, by pyroclastic materials of basaltic composition or by trachytes and pyroclastic material. Hydrudands have developed only in Pico Island (the youngest of the Archipelago), on pyroclastic materials of basaltic composition, occupying an area of 5500 ha, and showing very low bulk density, high water retention, quite high organic carbon content, high contents of Al, and extremely high phosphate adsorption capacity^124^. The young character of those soils might explain their shallowness and the smaller amount of accumulated carbon, when compared to the forest soils in the other two islands.

We found a tendency for a somewhat higher level of cations at EW soils, dominated by broad leaved species, what is to be expected since the uptake rates of acids and bases in broadleaf forests are higher than those in coniferous forests^125,126^.

Other studies have shown that the total stock of soil organic carbon varies among forest types^127,128^. For example, mixed conifer-hardwood systems have some of the largest soil carbon stocks in the USA^127,128^. In contrast, the mixed hardwood soils of the Northeast and northern Midwest of USA are formed on sandy substrates which have low surface area and consequently smaller soil carbon stocks which are very sensitive to disturbance^127,128^.

Soil textures in the three forest types included in this research varied widely from sandy, loam, to silt, reflecting complex landscape processes. However, there was a tendency for a dominance of sandy loam at the NF, sandy loam and loam at EW and PF, but with a lower content of loam in the latter. Also, the EW and PF at Pico showed a large contribution of sand or loamy sand. As seen above, this might be linked to the type of substrate available at Pico Island. The relatively high contribution of loam at EW in Terceira and São Miguel might also justify the relatively high content in cations mentioned above^129–131^.

## Conclusion

Regarding taxonomic diversity, there was ample evidence that NF harbored the highest plant diversity levels and PF the lowest, NF stands playing an important role as native plant diversity hotspots. Diversity levels were generally lower for structural than for taxonomic data, particularly for PF. Again, NF showed the highest levels of structural diversity, stressing their importance as plant diversity sinks, and eventually affecting other ecosystem functions such as water retention. Regarding carbon accumulation, total carbon stocks were mostly accumulated in tree biomass, with the largest values for PF and the lowest for NF, except for EW, in São Miguel Island, which showed extremely high values associated with old forest stands. PF provides economic assets and could play a role as carbon sink, while EW presently has a major role as a carbon sink, apparently also preserving soil quality. However, its role in biodiversity preservation is globally negative, and should be replaced by PF or NF where possible. Nonetheless, in future developments regarding a carbon market in the Azores, natural forests at low to medium elevations could be set as a priority to maximize carbon sequestration while preserving native biodiversity. Thus, our dataset and the derived conclusions will be useful for future conservation and research activities, as well as for forest managers, in the development of more comprehensive action plans, particularly on islands. Finally, we consider that this type of detailed scientific report, regarding carbon accumulation in different types of forests and shrubland, should be prioritized in relation to studies including only general estimates per forest type (e.g., tropical versus temperate forests). As shown here, variation among forests and sites, due to climatic, edaphic, dendrochronological, historical or management factors, might originate relevant differences in carbon accumulation estimates.

## Methods

### Site description

**Figure 2:**
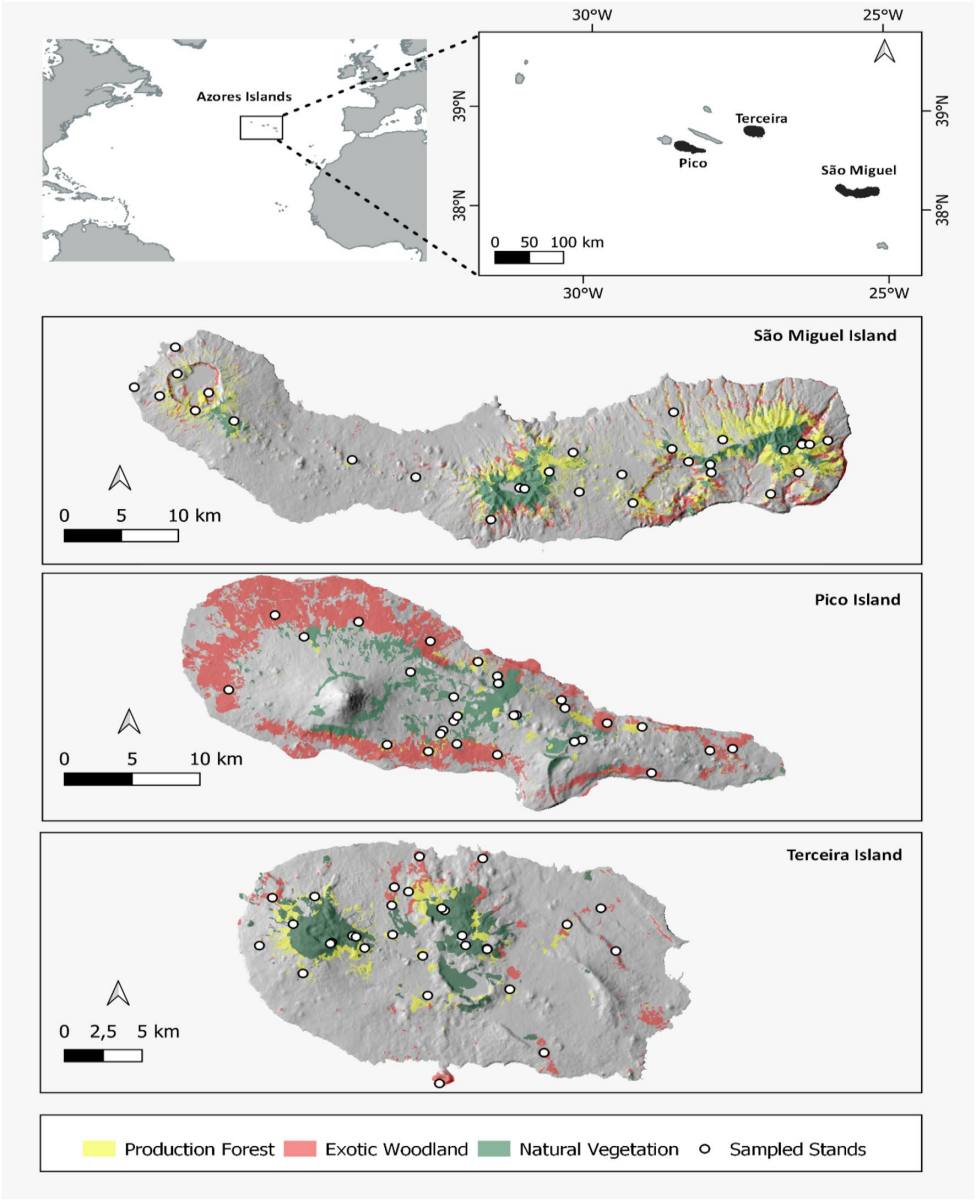
Location of the Azores archipelago (Portugal) and the distribution of the 90 selected stands, from each forest type (Natural Forest; Production Forest and Exotic Woodland) in the islands of São Miguel (30 stands), Terceira (30 stands) and Pico (30 stands). Figure edited by the authors using QGIS (version 2.18; http://qgis.org/), based on. Azorean Forest Inventory data^136^.

The study was conducted in the Azores archipelago (between 36°55 N and 39°42 N and 25°00 W and 31°30′ W), situated between North America and Europe, about 1500 km west of mainland Portugal. The archipelago has a total area of 2323 km^2^ and comprises nine inhabited islands of volcanic origin (Fig. 2). This research comprised three islands contributing with the largest forest areas: São Miguel Island with 745 km^2^, the highest elevation at 1,105 m a.s.l. and an estimated age of 0.79 MY (millions of years)^132^; Terceira Island with 400 km^2^, a maximum elevation of 1,023 m a.s.l. and 0.39 MY^133^ and; Pico Island with an area of 447 km^2^, mostly occupied by a volcano reaching an altitude of 2351 m a.s.l., and an approximate age of 0.27 MY^134^.

The three types of vegetation included in the study were: (i) Natural Forests, NF, submontane and montane cloud forests, characterized by geographic isolation, relatively homogeneous climatic conditions, a limited number of native woody species but high biodiversity and a high number of endemic species^60,61,92,135^, which are dominated by *Ilex azorica* Gand., *Juniperus brevifolia* (Hochst. ex Seub.) Antoine, *Laurus azorica* (Seub.) Franco, *Morella faya* (Aiton) Wilbur and *Picconia azorica* (Tutin) Knobl, relicts of once much more common formations; (ii) Exotic Woodland, EW, mostly dominated by *Pittosporum undulatum* Vent., a woody invader from Australia; and (iii) Production Forest, PF, including *Cryptomeria japonica* D.Don. Other species also present with lesser extent in PF are *Eucalyptus globulus* Labill. (mostly in Terceira) and *Pinus pinaster* Aiton (mostly in Pico)*.* Other types of vegetation common in the archipelago but that were not targeted in this study include intensive and extensive pasturelands, generally above 400 m in altitude, usually installed in sloped terrain where soils are more difficult to cultivate, and orchards and agricultural field crops located at lower elevations^136^. Quantitatively, pastures occupy 43% of the territory, PF represents 22%, NF 13% and EW 22%^49,61,73,95,137^.

*Pittosporum undulatum*, is the dominant woody species in the Azores, using about 30% of the forested area, i.e., 23,939 ha from a total of 49,343 ha occupied by forest in the archipelago^53,73,136^. This invasive species can overgrow the native vegetation by shading the indigenous species and forming pure stands, particularly in sheltered locations. Its introduction altered the natural transition between the native plant communities, which were found between 300 and 600 m of altitude^94^.

*Cryptomeria japonica* is considered the most important forestry species in the Azores archipelago, not only because of its economic importance, occupying 60% of the area dedicated to production forest, but also because its stands are a structural element of the Azorean landscapes^73,136^. Although, with a global distribution ranging from temperate to subtropical regions, major stands of *C. japonica* are found in subtropical conditions, in which the precipitation values reach 2540 mm annually, generally in pure stands and frequently on steep slopes, with abundant fogs and intense winds. Thus, in conditions similar to those found in the Azores at middle to high elevations^136^.

### Sampling and data collection

Field work was carried out on spring/summer of 2017 and 2018. A geographic information system (GIS; QGis 2.18) was used to map and identify forest stands, based on the data provided by the Azorean Forest Inventory^136^. A total of 90 forest stands were randomly sampled, 30 in each of the three selected islands São Miguel, Terceira and Pico (10 NF, 10 PF and 10 EW) (Fig. 2). At each forest we marked a plot with 10 × 10 m (100 m^2^), divided into 4 (5 × 5 m) subplots.

### Taxonomic diversity

We recorded vascular plants species namely gymnosperms, angiosperm, and ferns, within each subplot (5 × 5 m). Species that could not be identified with certainty in the field were collected, following standard herbarium techniques, and later identified at the AZB Herbarium using available literature^59,138,139^. A few dubious specimens were identified to genus level only. The abundance of each plant species was estimated based on the relative abundance according to cover (estimated visually at each subplot) and number of specimens within all the 360 subplots.

### Structural diversity

To access forest structure, a total of 2973 trees were recorded from each subplot (5 × 5 m) in all 90 plots, and the following dendrometric traits were measured: *H*, tree total height (m), using a Vertex IV 360° and Transponder T3, Haglöf Sweden AB; and *DBH,* diameter at breast height (cm), using a tree diameter measuring tape. Only individual trees and shrubs with *DBH* above 2.5 cm were included^53–55,140^. For individuals that branched at breast height or below, the diameter was measured, separately, at each branch.

### Leaf litter sampling

To characterize leaf litter biomass and carbon content, the leaf litter layer was collected using a 1 m^2^ frame, at the center of each subplot, in a total of 270 leaf litter samples. All coarse woody debris were removed from the samples prior to collection. The leaf litter samples were transported to the laboratory and weighed immediately, oven-dried at 60ºC for 48–72 h and reweighed. The bulk density (g of dry leaf per m^2^ of soil) was calculated and mean value per each 90 plots was used. A duplicate sample was collected and sent to the Soil and Plant Laboratory of the University of Trás-os-Montes and Alto Douro, in Vila Real, Portugal, to determine carbon content.

### Soil sampling

#### Bulk density

For soil bulk density, three subplots were selected randomly, at each of the 90 plots. At the center of each subplot, a 10-cm-depth trench was opened (or to depth at which impenetrable rocks were encountered) and undisturbed soil cores were collected using a volumetric ring with 1 mm of thickness, 8 cm of internal diameter and 10 cm of height. Mean values were used per site and a total of 270 soil samples, were taken. Samples were transported to the laboratory and weighted immediately, and then oven-dried at 60ºC for 48–72 h, and reweighted. The bulk density (g of dry soil per cm^3^ of soil) was calculated.

#### Soil parameters

To estimate soil parameters (organic carbon content, pH, macro/micronutrients, and soil texture), a total of 270 soil cores were collected with a soil sampler, taken randomly at each of the 90 plots, to collect the top 30 cm of soil (or to depth at which impenetrable rocks were encountered). Soil samples were sieved and sent to the Soil and Plant Laboratory of the University of Trás-os-Montes and Alto Douro, in Vila Real, Portugal, for analysis.

## Statistical analyses

### Species diversity

The most used representation of ecological diversity is species diversity, based on the number of species and on the relative abundance of each species found at a certain location^141^. We compared the mean diversity at each forest type using the Shannon index (H'), because it provides an account for both abundance and evenness (E’)^88^. It also does not disproportionately favor any species as it accounts all the species according to their frequencies^142^.

### Diversity partitioning

Diversity partitioning reveals the scale at which diversity is maximized^143^. The total species diversity/species richness of plants recorded in study sites were divided into diversity components (*α*, *β* and *γ*) at three spatial scales: *α*-diversity within plots, *β*-diversity defined as turnover of species among samples/plots at different localities, and *γ*-diversity for the whole region (number of species found in the pooled sampling units)^144^. At the lowest sampling level, *α*_*1*_ is the mean species diversity in a plot, *α*_2_ is the mean species diversity in a forest, *α*_3_ is the mean species diversity in a type of forest. As for *β*-diversity, *β*_*1*_ = (*α*_2_ − *α*_1_), *β*_*2*_ = (*α*_3_ − *α*_2_) and *β*_*3*_ = (*γ* − *α*_3_). Thus, the total diversity (*γ*) in the Azores forests can be partitioned as: *γ* = *α*_1_ + *β*_*1*_ + *β*_*2*_ + *β*_*3*_. Is this study *α* and *γ* diversities were measured directly, as numbers of species in the samples, at the different levels. These analyses were performed using the function “adipart” of the “vegan” R package^145^.

### Structural diversity

We included 2973 trees (*DBH* ≥ 2.5 cm) and reported the following forest parameters: tree density D (trees ha^−1^; number of individuals divided by sampled area); Maximum height, *H*; Diameter at breast height, *DBH* (recorded for of all the branches at breast height per tree); and Basal area, *BA* (m^2^; where *BA* = *DBH*^2^ × π/4, resulting from the sum of all branches per tree). *BA* is the cross-sectional area of woody stems, and it measures the relative dominance, that is, the degree of coverage of a species as an expression of the space it occupies in a forest. To estimate *BA* diversity among 90 plots, first we summed all basal areas from all trees of the same species per plot (*BA* per ha), and a mean value per plot was used to calculate different estimators: Shannon’s index, gamma diversity, and evenness. For tree maximum height, we used mean value per plot and standard deviation.

### Forest clustering

To identify patterns in the composition of Azorean forests we calculated Hellinger distance and Unweighted Paired Group Mean Average (UPGMA) as agglomeration schedule, since this was the combination originating the highest cophenetic correlation value between the community distance matrix and the dendrogram^146^. The optimal number of community groups was determined both by using silhouette widths, that is Rousseeuw quality index, and the optical number of clusters according to Mantel statistic (Pearson)^147^. We complemented this analysis with an ordination of the forest communities using all pairwise distances represented by a Non-Metric Multidimensional Scaling (NMDS), with the function “metaMDS” of the R “vegan” package, and the application of a PERMANOVA to determine the amount of variance on the vascular plant cover dataset, explained by forest type and island^148^.

### Indicator species

An analysis of the relative indicator species values^149^ was performed to determine the specificity (uniqueness to specific sites) and fidelity (rate of recurrence within each site) of each species regarding a particular forest. The indicator value method (IndVal) facilitates the identification of indicator species for a a priori established group of forests^150^. In this study the IndVal was based only on within-species comparisons of abundance and has been used to express the importance of species as ecological indicators in community classifications^147^. The level of significance was set at 0.05 and results from a permutation test. Species with significant indicator values above 70%^151,152^, were regarded as characteristic indicator species. Indicator Species Analyses were conducted by using the “multipatt2” function of the “Indicspecies” R package^153^.

### Carbon stock in trees

Aboveground biomass (*AGB*, kg expressed on a dry-weight basis) of each tree was estimated using allometric equations reported in the literature. Species-specific allometric equations were used for *P. undulatum, C. japonica, Clethra arborea, I. azorica, L. azorica, Morela faya, Phoebe indica* Pax*, Eucalyptus globulus* and *Acacia melanoxylon*^53,55,154–156^. For the remaining species generic allometric equations were used^157,158^. Depending on the species, biomass was estimated from *D*, *H*, *BA* and/or *NB* (number of branches; Supplementary Table S4 online)*.* Although belowground biomass (*BGB*, kg) in tree roots accounts for a large portion of the total forest biomass and provides an additional important carbon pool, there is still a lack of partitioning data for *BGB*. Thus, *BGB* of each tree was estimated using the standard ratio of root to shoot biomass for temperate oceanic forests, (see Table 4.4, Chapter 4,^159,160^). After calculating the total biomass (kg per tree) resulting from *AGB* + *BGB*, the carbon stocks (Mg C ha^−1^) per each forest type was estimated as follows: Biomass to carbon conversions were performed pursuant to the guidelines established in the IPCC Guidelines for National Greenhouse Gas Inventories, (see table 4.3, chapter 4,^159^), which assumes carbon content to be 48% for broad-leaved species and 51% for conifers species, of the *AGB* of each living tree^161–164^.

### Carbon stocks in soil and leaf litter

Total carbon stock in the soil (Mg ha^−1^) was estimated from organic matter (g kg^−1^) with conversion factor 0.58, so-called Van Bemmelen factor 1.724^165^, multiplied by bulk density (kg m^−3^), and considering the mean soil volume available per hectare, depending on soil depth at each site (5 to10 cm). Total leaf litter carbon (Mg ha^−1^) was estimated from dry biomass per sample (g dry litter m^−2^) and considering the amount of carbon by unit of litter biomass (g C kg^−1^ dry litter). For soil and leaf litter mean values were used per stand.

### Environmental variables

To compare climate on each forest type, we used mean annual temperature (TMEA), mean annual precipitation (PMEA), mean annual relative humidity (RHMEA) and altitude based on the CIELO Model^166^, a raster GIS environment with 100 m spatial resolution that is used to model local scale climate variables relying on limited available data from synoptic coastal meteorological stations^167^. The CIELO model has been calibrated and validated to the Azorean islands and is available through CLIMAAT project (https://www.climaat.angra.uac.pt) and in Azevedo & Pereira^166,168^.

### Statistical tests

Comparisons of all the parameters between forest types and islands was undertaken using two-way ANOVA followed by a post-hoc Tukey HSD test, after verification of the assumptions of their application (i.e., normality, homoscedasticity). Statistical analysis was performed with IBM Corp. Released 2019. IBM SPSS Statistics for Windows, Version 26.0. Armonk, NY: IBM Corp.

## Supplementary Information

Supplementary Information.
