# Supplementary material for: Taxonomic, structural diversity and carbon stocks in a gradient of island forests
Source: Sci Rep. 2022 Jan 20;12:1038. doi: 10.1038/s41598-022-05045-w (PMC8776957; doi:10.1038/s41598-022-05045-w)
Supplement: Supplementary file 1 — Supplementary Information. [file 41598_2022_5045_MOESM1_ESM.docx]

**Supplementary information**

**Taxonomic, structural diversity and carbon stocks in a gradient of island forests**

Lurdes Borges Silva ^(1,2*)^, Diogo C. Pavão^(1,2)^, Rui B. Elias^(3,4)^, Mónica Moura^(1,2)^, Maria A. Ventura^(1,2)^, Luís Silva^(1,2)^

^(1)^ CIBIO, Centro de Investigação em Biodiversidade e Recursos Genéticos, InBIO Laboratório Associado, Pólo dos Açores, Universidade dos Açores, Campus de Ponta Delgada, Rua da Mãe de Deus, 9500-321 Ponta Delgada, Açores, Portugal.

^(2)^ Faculdade de Ciências e Tecnologia, Universidade dos Açores, Campus de Ponta Delgada, Rua da Mãe de Deus, 9500-321 Ponta Delgada, Açores, Portugal.

^(3)^ CE3C/ABG – Centre for Ecology, Evolution and Environmental Changes/Azorean Biodiversity Group, Universidade dos Açores, Campus de Angra do Heroísmo, Rua Capitão João d’Ávila – Pico da Urze, 9700-042 Angra do Heroísmo.

^(4)^ Faculdade de Ciências Agrárias e do Ambiente, Universidade dos Açores, Campus de Angra do Heroísmo, Rua Capitão João d’Ávila – Pico da Urze, 9700-042 Angra do Heroísmo.

*Corresponding author: (lurdes.cb.silva@uac.pt)

**Supplementary Table S1.** Indicator species analysis. Species that had an indicator value (IndVal>70) and were significant at (P< 0.05), are listed in order by descending indicator value within each plant community type.

| Forest | Taxa | IndVal |
| --- | --- | --- |
| Natural forest | *Vaccinium cylindraceum* | 0.95 |
|  | *Myrsine africana* | 0.95 |
|  | *Ilex perado ssp. azorica* | 0.93 |
|  | *Laurus azorica* | 0.89 |
|  | *Lysimachia azorica* | 0.88 |
|  | *Culcita macrocarpa* | 0.87 |
|  | *Hymenophyllum tunbrigense* | 0.85 |
|  | *Luzula purpureosplendens* | 0.84 |
|  | *Struthiopteris spicant* | 0.83 |
|  | *Juniperus brevifolia* | 0.82 |
|  | *Athyrium felix femina* | 0.80 |
|  | *Selaginella Kraussiana* | 0.80 |
|  | *Dryopteris aemula* | 0.79 |
|  | *Elaphoglossum semicylindricum* | 0.76 |
|  | *Frangula azorica* | 0.74 |
|  | *Pteridium aquilinum* | 0.73 |
|  | *Erica azorica* | 0.70 |
| Exotic woodland | *Pittosporum undulatum* | 0.93 |
|  | *Morella faya* | 0.73 |
| Production Forest | *Cryptomeria japonica* | 0.99 |

**Supplementary Table S2**. Comparison of soil properties at 90 forests in the Azores, from three islands (Pico, São Miguel, and Terceira) and three forest types (Exotic Woodland, Natural Forest, and Production Forest). Results of a two-way ANOVA. Bold indicates a significant effect (p<0.05).

|  | Type | |  | Island | |  | Interaction | |
| --- | --- | --- | --- | --- | --- | --- | --- | --- |
| Parameter | F | p |  | F | p |  | F | p |
| pH | 17.4 | **0.000** |  | 13.2 | **0.000** |  | 1.3 | 0.284 |
| P\|ER | 6.4 | **0.003** |  | 5.9 | **0.004** |  | 1.8 | 0.135 |
| K\|ER | 5.3 | **0.007** |  | 14.8 | **0.000** |  | 0.2 | 0.953 |
| P\|O | 4.5 | **0.014** |  | 5.8 | **0.004** |  | 2.0 | 0.107 |
| Ca^2+^ | 7.2 | **0.001** |  | 10.3 | **0.000** |  | 3.0 | **0.027** |
| Mg^2+^ | 4.0 | **0.021** |  | 13.0 | **0.000** |  | 4.4 | **0.003** |
| K^+^ | 5.1 | **0.008** |  | 2.2 | 0.117 |  | 1.3 | 0.280 |
| Na^+^ | 0.6 | 0.574 |  | 37.7 | **0.000** |  | 1.1 | 0.386 |
| Al+ H3O^+^ | 9.6 | **0.000** |  | 17.0 | **0.000** |  | 2.7 | **0.037** |
| Electrical conductivity | 2.8 | 0.068 |  | 26.4 | **0.000** |  | 4.3 | **0.004** |
| N total | 1.3 | 0.287 |  | 56.3 | **0.000** |  | 1.8 | 0.131 |
| Coarse sand | 1.8 | 0.177 |  | 116.0 | **0.000** |  | 3.3 | **0.015** |
| Fine sand | 7.6 | **0.001** |  | 47.2 | **0.000** |  | 1.5 | 0.220 |
| Silt | 3.5 | **0.034** |  | 135.9 | **0.000** |  | 1.8 | 0.144 |
| Clay | 4.6 | **0.013** |  | 15.642 | **0.000** |  | 6.4 | **0.000** |

**Supplementary Table S3**. Soil variables found at 90 forests in the Azores, from three islands (Pico, São Miguel, and Terceira) and three forest types (Exotic Woodland, Natural Forest, and Production Forest). Mean (m) and standard error (se) for each environmental parameter. For each row, different letters indicate significant differences (p<0.05) according to the results of a Tukey test applied after ANOVA.

| **Parameter** |  |  | **Exotic Woodland** | | | | | |  | **Natural Forest** | | | | | |  | **Production Forest** | | | | | |
| --- | --- | --- | --- | --- | --- | --- | --- | --- | --- | --- | --- | --- | --- | --- | --- | --- | --- | --- | --- | --- | --- | --- |
|  |  |  | **Pico** | | **São Miguel** | | **Terceira** | |  | **Pico** | | **São Miguel** | | **Terceira** | |  | **Pico** | | **São Miguel** | | **Terceira** | |
| **BD** | m |  | 0.2 | a | 0.6 | c | 0.6 | c |  | 0.1 | a | 0.5 | c | 0.4 | bc |  | 0.2 | ab | 0.6 | c | 0.4 | c |
| (g.cm^-3^) | se |  | 0.0 |  | 0.1 |  | 0.1 |  |  | 0.0 |  | 0.0 |  | 0.1 |  |  | 0.0 |  | 0.0 |  | 0.0 |  |
| **OM** | m |  | 286.3 | c | 142.0 | ab | 168.5 | abc | | 409.2 | d | 118.4 | a | 165.5 | ab |  | 247.5 | bc | 109.5 | a | 174.0 | abc |
| (g.kg^-1^) | se |  | 33.7 |  | 13.4 |  | 26.9 |  |  | 48.4 |  | 20.1 |  | 21.3 |  |  | 22.3 |  | 11.4 |  | 21.0 |  |
| **N** | m |  | 11.8 | c | 5.9 | a | 6.6 | ab |  | 13.6 | c | 4.0 | a | 6.6 | ab |  | 10.4 | bc | 4.2 | a | 6.7 | ab |
| (g.kg^-1^) | se |  | 1.3 |  | 0.7 |  | 0.9 |  |  | 1.3 |  | 0.4 |  | 0.8 |  |  | 0.8 |  | 0.5 |  | 0.7 |  |
| **pH H_2_O** | m |  | 5.7 | bcd | 6.1 | d | 5.6 | bcd | | 5.0 | a | 5.5 | abc | 5.2 | ab |  | 5.8 | cd | 6.1 | d | 5.4 | abc |
|  | se |  | 0.1 |  | 0.1 |  | 0.2 |  |  | 0.2 |  | 0.1 |  | 0.1 |  |  | 0.1 |  | 0.1 |  | 0.2 |  |
| **P\|ER** | m |  | 17.4 | ab | 7.0 | a | 21.2 | ab |  | 34.2 | c | 8.4 | a | 26.5 | ab |  | 10.8 | a | 7.7 | a | 6.9 | a |
| (mg.kg^-1^) | se |  | 3.9 |  | 1.6 |  | 4.8 |  |  | 9.2 |  | 2.6 |  | 8.3 |  |  | 3.4 |  | 2.8 |  | 1.6 |  |
| **P\|O** | m |  | 12.7 | a | 15.8 | ab | 27.8 | ab |  | 29.8 | ab | 10.9 | a | 37.7 | b |  | 11.2 | a | 12.9 | a | 16.5 | ab |
| (mg. kg^-1^) | se |  | 2.3 |  | 2.2 |  | 8.2 |  |  | 7.3 |  | 3.0 |  | 7.9 |  |  | 1.8 |  | 3.0 |  | 5.2 |  |
| **K\|ER** | m |  | 189.1 | abc | 396.9 | b | 169.7 | abc | | 139.9 | ab | 277.4 | bc | 59.1 | ab |  | 75.2 | ab | 240.7 | abc | 46.1 | a |
| (mg.kg^-1^) | se |  | 24.9 |  | 108.5 |  | 19.2 |  |  | 20.7 |  | 85.2 |  | 5.9 |  |  | 10.2 |  | 51.0 |  | 3.8 |  |
| **K^+^** | m |  | 0.6 | a | 1.2 | a | 1.1 | a |  | 0.8 | a | 0.7 | a | 0.3 | a |  | 0.3 | a | 0.7 | a | 0.3 | a |
| (cmol_c_.kg^-1^) | se |  | 0.1 |  | 0.4 |  | 0.4 |  |  | 0.1p |  | 0.2 |  | 0.1 |  |  | 0.0 |  | 0.1 |  | 0.1 |  |
| **Ca^2+^** | m |  | 11.4 | c | 5.7 | abc | 9.5 | bc |  | 11.5 | c | 3.4 | ab | 2.7 | a |  | 5.6 | abc | 4.8 | ab | 3.1 | ab |
| (cmol_c_.kg^-1^) | se |  | 2.0 |  | 1.1 |  | 2.2 |  |  | 2.7 |  | 0.5 |  | 0.4 |  |  | 0.8 |  | 0.9 |  | 0.4 |  |
| **Mg^2+^** | m |  | 6.7 | abc | 3.4 | ab | 8.5 | bc |  | 10.3 | c | 2.1 | a | 2.4 | a |  | 5.5 | abc | 2.1 | a | 2.7 | a |
| (cmol_c_.kg^-1^) | se |  | 1.0 |  | 0.5 |  | 1.6 |  |  | 2.7 |  | 0.4 |  | 0.5 |  |  | 0.9 |  | 0.3 |  | 0.6 |  |
| **Na^+^** | m |  | 1.1 | bc | 0.7 | ab | 0.5 | a |  | 1.3 | c | 0.4 | a | 0.2 | a |  | 1.2 | bc | 0.5 | a | 0.3 | a |
| (cmol_c_.kg^-1^) | se |  | 0.2 |  | 0.1 |  | 0.2 |  |  | 0.1 |  | 0.1 |  | 0.1 |  |  | 0.2 |  | 0.1 |  | 0.1 |  |
| **Al ^+^ H3O^+^** | m |  | 0.2 | a | 0.1 | a | 0.2 | a |  | 0.4 | abc | 0.2 | ab | 0.6 | c |  | 0.1 | a | 0.0 | a | 0.6 | bc |
| (cmol_c_.kg^-1^) | se |  | 0.0 |  | 0.0 |  | 0.1 |  |  | 0.1 |  | 0.1 |  | 0.1 |  |  | 0.0 |  | 0.0 |  | 0.2 |  |

**
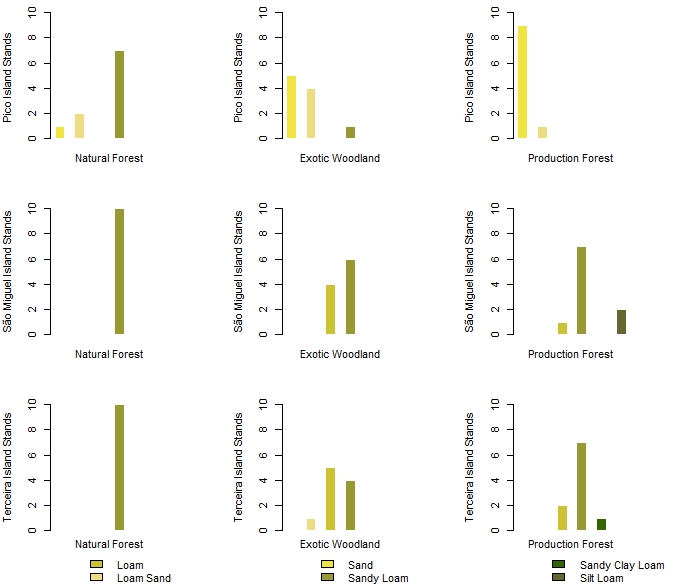
**

**Supplementary Figure S1.** Characterization of soil texture per each forest type (90 stands) and for the three islands.

**Supplementary Table S4.** Allometric equations used to estimate *AGB* (kg) and *BGB* (kg), were based on diameter at breast height, *DBH* (cm, resulting from the sum of all branches), tree height, *H* (m); number of branches at breast height (1.30 m), *NB*; and basal area, *BA* (cm^2^; where *BA* = *DBH*^2^ x π/4, resulting from the sum of all branches per tree). Note: *a*, *b*, are scaling coefficients that vary with the variables under investigation.

| **Species** | **Allometric equation** | **References** |
| --- | --- | --- |
| *Acacia melanoxlon* | ln(*AGB*)= a`+b_1_ ln(*DBH*) + ε | [1] |
| *Banksia integrifolia* | ln(*AGB*)= ln(a)+b_1_ ln(*DBH*) | [2] |
| *Cletra arborea* | ln(*AGB*)= a + ln(*DBH*^b^) + ε | [3] |
| *Cryptomeria japonica* | ln(*AGB*+*BGB*)= a + b_1_ ln (*DBH*) + b_2_ ln (*H*) | [4] |
| *Erica azorica* | ln(*AGB*) = a +b_1_ ln(*DBH*) + ε | [5] |
| *Eucalyptus globulus* | ln(*AGB*)= a`+b_1_ ln(*DBH*) + ε | [1] |
| *Euphorbia stygiana* | ln(*AGB*) = a +b_1_ ln(*DBH*) + ε | [6] |
| *Frangula azorica* | ln(*AGB*) = a +b_1_ ln(*DBH*) + ε | [6] |
| *Ilex perado* subsp. a*zorica* | ln(*AGB*) = a +b_1_ ln(*DBH*) + ε | [5] |
| *Juniperus brevifolia* | ln(*AGB*) = a + ln(*DBH*^b^) + ε | [3] |
| *Laurus azorica* | ln(*AGB*) = a +b_1_ ln(*DBH*) + ε | [5] |
| *Laurus nobilis* | ln(*AGB*) = a +b_1_ ln(*DBH*) + ε | [5] |
| *Myrsine africana* | ln(*AGB*) = a +b_1_ ln(*DBH*) + ε | [6] |
| *Morella faya* | ln(*AGB*) = a +b_1_ ln(*DBH*) + ε | [5] |
| *Ocotea foetens* | ln(*AGB*) = a +b_1_ ln(*DBH*) + ε | [6] |
| *Picconia azorica* | ln(*AGB*) = a +b_1_ ln(*DBH*) + ε | [6] |
| *Pittosporum undulatum* | ln(*AGB*)= a + b_1_ ln(*BA^2^H*) + b_2_ ln(*DBH^2^H*) + b_3_ ln(*H^2^*) + b_4_ ln( *NB^2^*) + ε | [7] |
| *Psidium littorale* | ln(*AGB*) = a + ln(*DBH*^b^) + ε | [3] |
| *Ulmus procera* | (*AGB*)=0.0044*DBH*^2.438^+0.0068*DBH*^3.001^+0.1308*DBH*^2.271^ | [8] |
| *Viburnum treleasei* | ln(*AGB*) = a +b_1_ ln(*DBH*) + ε | [6] |

**References**

1. Paul, K.I. *et al*. Development and testing of allometric equations for estimating above-ground biomass of mixed-species environmental plantings. *For. Ecol. Manage*. **310**, 483-494 <https://doi.org/10.1016/j.foreco.2013.08.054> (2013).
2. Zianis, D. & Mencuccini, M. On simplifying allometric analyses of forest biomass. *For. Ecol. Manage*. **187**, 311-332 <https://doi.org/10.1016/j.foreco.2003.07.007> (2004).
3. Rojas-García, F., De Jong, B.H.J., Martínez-Zurimendí, P. & Paz-Pellat, F. Database of 478 allometric equations to estimate biomass for Mexican trees and forests. *Ann. For. Sci.* **72**, 835-864 <https://doi.org/10.1007/s13595-015-0456-y> (2015).
4. Lim, K.H., Lee, K-H., Lee, K.H, & Park, I.H. Biomass expansion factors and allometric equations in an age sequence for Japanese cedar (*Cryptomeria japonica*) in southern. *J. For. Res*. **18**, 316-322 <https://doi.org/10.1007/s10310-012-0353-2> (2013).
5. Aboal, J., Arévalo, J.R. & Fernández, Á. Allometric relationships of different tree species and stand above ground biomass in the Gomera laurel forest (Canary Islands). *Flora*. **200**, 264-274 <https://doi.org/10.1016/j.flora.2004.11.001> (2005).
6. Fernández-Palacios, J.M., Esteban, G.J.J., Lopez R.J. & Luzardo, M.C. Approach to the assesment of aereal biomass and net primary production in a laurel forest station on Tenerife. *Vieraea*. **20**, 11–20 (1991).
7. Borges Silva, L. *et al*. Biomass valorization in the management of woody plant invaders: the case of *Pittosporum undulatum* in the Azores. *Biomass Bioenergy*. **109**, 155-165 <https://doi.org/10.1016/j.biombioe.2017.12.025> (2018).
8. He, H. *et al*. Allometric biomass equations for 12 tree species in coniferous and broadleaved mixed forests, Northeastern China. *PLoS ONE*. **13**, e0186226 <https://doi.org/10.1371/journal.pone.0186226> (2018).
